# Vitamin D and follicular recruitment in the in vitro fertilization cycle

**DOI:** 10.5935/1518-0557.20240005

**Published:** 2024

**Authors:** Roberto de A. Antunes, Brenda M. L. de Melo, Maria do Carmo B. de Souza, Marcelo M. de Souza, Gabriela P. S. Melo, Thamires F. M. Jandre, Ana Cristina A. Mancebo, Flavia L. Conceição, Tania M. Ortiga-Carvalho

**Affiliations:** 1 Gynecology Department of the Clementino Fraga Filho University Hospital - Federal University of Rio de Janeiro - Rio de Janeiro - RJ, Brazil; 2 Fertipraxis - Human Reproduction Center, Rio de Janeiro - RJ, Brazil; 3 Endocrinology Department of the Clementino Fraga Filho University Hospital Federal University of Rio de Janeiro - Rio de Janeiro - RJ, Brazil; 4 Translational Endocrinology Lab of the Carlos Chagas Filho, Biophysics Institute - Federal University of Rio de Janeiro - Rio de Janeiro - RJ, Brazil

**Keywords:** vitamin D, infertility, *in vitro* fertilization, follicular development

## Abstract

**Objective:**

Vitamin D (VD) is a fat-soluble steroid hormone, synthesized by the skin, most known for its role in bone mineral balance. Vitamin D receptors (VDR) are also found in the female reproductive system, but their role remains unclear. The objective of this study was to analyze the relationship between serum vitamin D levels and the number of oocytes retrieved after ovarian stimulation.

**Methods:**

This is a retrospective study involving 267 patients undergoing in vitro fertilization (IVF) carried out in the Fertipraxis clinic, a private practice facility. The patients were initially divided into two groups according to their VD levels. Group 1 included 152 patients with VD levels < 30 ng/mL and group 2 had 115 patients with VD levels > 30 ng/mL. They were further analyzed and separated considering their age, anthropometric data, ovarian reserve, amount of gonadotropin used, and follicles obtained until trigger day.

**Results:**

In our analysis, there were no difference in the number of follicles and oocytes retrieved, nor in the number of mature oocytes obtained from patients with both vitamin D deficiency and sufficiency.

**Conclusions:**

The results of our study show no difference among number of follicles, oocytes retrieved and mature oocytes obtained after ovarian stimulation according to their vitamin D serum levels. Further higher-quality studies are needed to evaluate the possible roles of serum vitamin D levels in other stages of human fertilization process.

## INTRODUCTION

Vitamin D (VD) is a fat-soluble steroid hormone, synthesized by the skin, known for its role in calcium and phosphorus metabolism, and bone remodeling. It is essential to maintain bone mineral balance (Irani & Merhi, 2014; Rehman *et al*., 2021; Tian *et al*., 2022). Its most common form in circulation is 25-hydroxy-vitamin D (25OH-D), synthesized by the liver, then metabolized in the kidneys by the 1-alpha-hydroxylase enzyme into its active form, 1,25-dihydroxy-vitamin-D (1,25(OH)2D) (Irani & Merhi, 2014; Hornstein, 2019; Makieva *et al*., 2021). 1,24(OH)2D acts through its binding to the vitamin D receptor (VDR) (Rehman *et al*., 2021; Tian *et al*., 2022, Karimi *et al*., 2021).

Even though VD classical actions are described in the calcium-phosphate metabolism, the presence of VDR and enzyme 1 alpha-hydroxylase in the endometrium, ovaries (granulosa cells and cumulus oophorous), placenta, hypothalamus, and pituitary gland indicate that VD might play a part in human reproduction. Several studies have been carried out to elucidate the role of VD in human reproduction, suggesting its relationship with spermatogenesis, ovarian reserve, follicular development, embryonic implantation, endometrial thickness, and its correlation with in vitro fertilization (IVF) (Rehman *et al*., 2021; Tian *et al*., 2022; Hornstein, 2019; Neville *et al*., 2016). In addition, other studies suggest that VD may influence the anti-Müllerian hormone (AMH) activity through its 25OH-D form, which would act on AMH type II receptors (AMHR-2) to inhibit the recruitment and maturation of follicles (Hornstein, 2019; Muscogiuri *et al*., 2017; Skowrońska *et al*., 2022; Voulgaris *et al*., 2017). Hypovitaminosis D is correlated with insulin resistance, polycystic ovary syndrome (PCOS) and endometriosis (Tian *et al*., 2022; Neville *et al*., 2016; Dastorani *et al*., 2018; Cunningham *et al*., 2019).

VD deficiency is frequently found in women at reproductive age, affecting more than 123 million women and 15% of the infertile population worldwide (Cozzolino *et al*., 2020; Fernando *et al*., 2020; Antunes *et al*., 2018). It can be defined as levels below 20 ng/mL, while insufficiency is when those levels are between 20 and 30 ng/mL (Irani & Merhi, 2014). Although, it is important to emphasize that these definitions were obtained from data of VD effects in bone remodeling. VD role in reproduction is still controversial, requiring more studies on the subject (Abadia *et al*., 2016; Lv *et al*., 2016). In our study, we used 30 ng/mL as parameter for the division between the groups, as it is the value referred by the Endocrine Society of North America (Irani & Merhi, 2014).

## MATERIALS AND METHODS

Retrospective study of 267 patients, between 29 and 40 years old, undergoing IVF at the Fertipraxis clinic between January 2017 and December 2020. The patients were divided into 2 groups. Group 1 (G1) is made up of 152 patients with insufficient or deficient vitamin D levels (less than 30 ng/mL). Group 2 (G2), has 115 patients with normal levels of vitamin D (greater than ≥ 30 ng/mL).

All our VD plasma level samples were obtained together with the couple’s blood tests for the required serologies according to the Brazilian regulatory norms for IVF. Therefore, all VD evaluations were collected up to three months from the beginning of the stimulation cycles. The patients that were supplementing VD, kept on doing so without changes in their supplementation protocol. No patients started new VD supplementation based on the presented VD levels.

Ovarian stimulation was performed with combinations of FSH and LH (Menopur^®^, Ferring Switzerland and Pergoveris^®^, Merck, Germany) ranging from 150UI to 300UI a day, according to clinical demands. Pituitary blockage was achieved using Cetrorelix Acetate (Cetrotide®, Merck, Germany), initiated when the largest follicle reached 14mm.

Initially, we ran a descriptive analysis of vitamin D, oocytes retrieved, M2 mature oocytes by the end of stimulation, follicles ≥ 15mm, follicles between 10-14 mm, total FSH administered, stimulus duration, presence of endometriosis, BMI, AMH level and age.

Subsequently, 6 Generalized Linear Models using the Gamma distribution were run to test the impact of vitamin D on the variables mentioned above. The Gamma distribution was chosen because the outcome variables were not normally distributed according to the Kolmogorov-Smirnov test. The analysis was performed using the SPSS for Windows version 23 software.

The primary objective of this study was to compare the number of oocytes retrieved and mature oocytes among both groups. The secondary objective was the analysis of the difference between duration of stimulation, quantity of gonadotropins used and follicular size.

## RESULTS

Group 1 had an average plasma VD level of 23.1 ng/mL (ranging from 11.0ng/mL - 29.9ng/mL); while Group 2 had 39.08ng/mL (range 30.0ng/mL - 66.4ng/mL). Table 1 presents the descriptive results of the continuous variables.

**Table 1:** Descriptive results of continuous variables.

Parameter	Total				Group 1 (vitamin D < 30 ng/mL)				Group 2 (vitamin D ≥ 30 ng/mL)				K-S
	**Min**	**Max**	**M**	**SD**	**Min**	**Max**	**M**	**SD**	**Min**	**Max**	**M**	**SD**	
Vitamin D	--	--	--	--	11.0	29.9	23.1	--	30.0	66.4	39.8	--	--
Age	28	40	36.43	2.82	29	40	36.68	2.65	28	40	36.10	3.02	0.159^***^
BMI	17.40	44.60	24.73	5.88	0	41.20	25.04	5.53	0	44	24.31	6.32	0.131^***^
AMH	0.02	12	2.01	2.08	0	12	2.09	2.20	0	11.32	1.88	1.89	0.180^***^
Stimulation duration	6	17	9.98	1.82	0	17	9.87	1.87	6	16	10.11	1.75	0.202^***^
Total UI FSH	10	4950	2207.38	851.26	840	4950	2278.64	864.88	10	4650	2119	829	0.098^***^
Follicles 10-14 mm	0	26	7.54	4.75	0	26	7.31	4.82	1	26	7.84	4.66	0.122
Follicles ≥ 15 mm	0	19	4.38	3.96	0	19	4.34	4.02	0	16	4.43	3.88	0.169^***^
Number of oocytes retrieved	0	44	9.38	7.05	0	32	9.03	6.67	0	44	9.81	7.51	0.105^***^
M2	0	27	7.09	5.26	0	27	6.80	5.08	0	25	7.46	5.49	0.121^***^

n = number of participants; Min = minimum score; Max = maximum score, M = mean; SD = standard deviation; K-S = Kolmogorov-Smirnov Test;

*** = *p*< .001.

Women in both groups had similar age and anthropometric characteristics (BMI in G1: 25.04±5.53 x BMI in G2: 24.31±6,32), as well as stimulation time and dosage of gonadotropins used. G1 showed slightly higher AMH levels (2.09) compared to G2 (1.88), but that it was not statistically significant (*p*<0.18) in our study. The number of recruited follicles was consistent between both groups. Among those ranging from 10 to 14mm, G1 had an average of 7.31 and G2 7.84 (*p*<0.122). When evaluating those greater than 15mm, G1 had an average of 4.34, while G2 had an average of 4.43 (*p*<0.169). There were no differences between the numbers of recovered oocytes (G1 9.03 x G2 9.81 - *p*<0.105) and MII oocytes by the end of the stimulation (G1 6.80 x G2 7.46 - *p*<0.121). Table 2 presents the results of the six Generalized Linear Models (Gamma distribution) that tested the impact of vitamin D on the outcome variables previously described. As we can see, vitamin D was not a predictor variable in any model tested (*p*>.05).

**Table 2:** Results of Generalized Linear Models tested (Gamma distribution).

Model	Predictors	_______ Exp (B)	Wald’s chi-square	p	Chi-square	p
**Model 1** (Number of oocytes retrieved)	Constant	23.49	27.75	.000	69.78(5)	.000
	Vitamin D	0.933	0.615	.433		
**Model 2 (M2)**	Constant	19.651	23.24	.000	59.58(5)	.000
	Vitamin D	0.941	0.440	.507		
**Model 3** (Follicles ≥ 15mm)	Constant	19.81	25.194	.000	64.60(5)	.000
	Vitamin D	0.972	0.092	.761		
**Model 4** (Follicles 10-14 mm)	Constant	9.217	17.629	.000	44.123(5)	.000
	Vitamin D	0.952	0.403	.526		
**Model 5** (Total UI FSH)	Constant	289.823	802.478	.000	23.212(5)	.000
	Vitamin D	0.944	1.058	.331		
**Model 6** (Duration of stimulation)	Constant	205.703	8.877	.000	14.519(5)	.013
	Vitamin D	1.009	0.977	.315		

All models presented were controlled by the presence or absence of endometriosis, BMI, AMH and age.

Chi-square = chi-square of the likelihood ratio; *p* = statistical significance.

Considering that the Chi-square of the General Model was statistically significant, evidencing the possibility that the control variables had had a significant impact on the outcomes. Table 3 shows the results of the tested control variables, considering the β-regression coefficient of each model.

**Table 3:** Results of control variables tested.

Model	Control variables	Wald’s chi-square	p	β	Exp (B)	95% CI for Exp (B)	
						Inf	Sup
Model 1 (Number of oocytes retrieved)	Endometriosis	0.004 (1)	.948	-0.008	0.993	0.792	1.244
	Age	5.846 (1)	.016	-0.038	0.963	0.934	0.993
	BMI	0.328 (1)	.567	0.004	1.004	0.990	1.019
	AMH	44.205 (1)	.000	0.169	1.184	1.127	1.245
Model 2 (M2 Ooocytes)	Endometriosis	0.501 (1)	.479	-0.083	0.920	0.731	1.159
	Age	5.798 (1)	.016	-0.038	0.963	0.933	0.993
	BMI	0.079 (1)	.779	0.002	1.002	0.987	1.018
	AMH	36.692 (1)	.000	0.154	1.167	1.110	1.227
Model 3 (Follicles ≥ 15mm)	Endometriosis	0.074 (1)	.786	-0.033	0.764	0.764	1.225
	Age	13.798 (1)	.000	-0.059	0.914	0.914	0.972
	BMI	5.214 (1)	.022	0.016	1.002	1.002	1.030
	AMH	37.019 (1)	.000	0.157	1.170	1.113	1.231
Model 4 (Follicles 10-14 mm)	Endometriosis	1.223 (1)	.726	-0.036	0.964	0.786	1.182
	Age	1.223 (1)	.269	-0.015	0.985	0.959	1.012
	BMI	0.243 (1)	.622	0.003	1.003	0.990	1.016
	AMH	33.361 (1)	.000	0.133	1.143	1.092	1.196
Model 5 (Total UI FSH)	Endometriosis	1.583 (1)	.208	0.098	1.103	0.947	1.284
	Age	4.367 (1)	.037	0.022	1.022	1.001	1.043
	BMI	4.657 (1)	.031	0.011	1.011	1.001	1.021
	AMH	14.031 (1)	.000	-0.053	0.948	0.922	0.975
Model 6 (Duration of stimulation)	Endometriosis	1.547 (1)	.214	0.038	1.038	0.979	1.102
	Age	0.043 (1)	.836	0.001	1.001	0.993	1.009
	BMI	7.200 (1)	.007	0.005	1.005	1.001	1.009
	AMH	6.429 (1)	.011	-0.014	0.986	0.976	0.997

*p* = statistical significance; inf = inferior limit; sup = superior limit.

Furthermore, a Spearman correlation analysis was performed between vitamin D levels and possible outcomes, in addition to other variables such as age, BMI and AMH. The choice for a non-parametric analysis was made because the variables did not have a normal distribution (Table 4). The cutoff points used for interpreting the correlation coefficients: 0.00 - 0.10 = negligible correlation; 0.11 - 0.29 = weak correlation; 0.30 - 0.49 = moderate correlation and > 0.50 = strong correlation (Cohen, 1988).

**Table 4:** Correlation analysis results.

		Vitamin D	Oocytes retrieved (n)	M2	≥ 15mm	10-14mm	Total UI FSH	Duration of stimulation	Age	BMI	AMH
Vitamin D	*p*	1	.065	.073	.016	.064	-.072	.031	-.100	-.070	-.001
	*p*		.132	.089	.720	.138	.102	.494	.023	.095	.975

*p* = Spearman’s rho; *p* = statistical significance.

**Figure 1:**
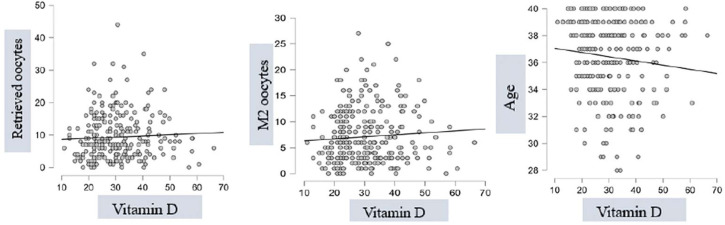
Spearman Correlation on Vitamin D x Retrieved Oocytes; Vitamin D x M2 oocytes; Vitamin D x Age.

Vitamin D levels also did not correlate with any of the analyzed variables, except for a significant inverse correlation with age in the study group (Figure 1).

Among the causes of infertility, the most common one in both groups was the male factor, followed by the ovarian factor in G1 and alterations in the female anatomy in G2. Only 2 patients in the first group and 3 in the second group had PCOS.

## DISCUSSION

Vitamin D has been discussed as a possible modifiable factor related to infertility (Gaskins & Chavarro, 2018). There are receptors for it in the female reproductive system, but its function is still unclear (Franasiak *et al*., 2017; Fichera *et al*., 2020; Somigliana *et al*., 2021). Among the possible actions of VD in reproduction, we can highlight its role in folliculogenesis and oocyte maturation, in addition to embryonic implantation (Fichera *et al*., 2020; Grzeczka *et al*., 2022). In our study we tried to determine if there is an actual correlation between plasma levels of vitamin D, follicular growth and oocyte maturity rates. However, our data shows no such correlation.

Neville *et al*. (2016) evaluated 73 men and 64 women, assessing the role of VD in both male and female reproductive systems. The study found no association between VD levels and each partner’s variables for IVF outcomes. In our study, a higher rate of recovered oocytes was found among patients with normal VD levels, but without statistical significance.

Banker *et al*. (2017) analyzed 291 patients, including egg donors and recipients, without finding better oocyte quality results or greater endometrial receptivity in subgroups with replete VD.

Abadia *et al.* (2016) in their analysis of 100 women undergoing IVF subdivided its study population in quartiles according to their 25OH-D status and also found no difference in the number of retrieved M2 oocytes in each group. They found a significant correlation between fertilization rates and higher 25OH-D levels; however, they have not analyzed the partner’s VD status which might prove to be an important confounder. They also did not find any correlation with clinical pregnancy rates or live birth rates (Abadia *et al*., 2016).

In their study, Iliuta *et al*. (2022) analyzed 15 articles from 9 different countries and found no relationship between normal VD levels, the number of oocytes retrieved and implantation rates. However, there was statistical significance between VD repletion and biochemical pregnancy, ongoing pregnancy, and live birth rate (Wu *et al*., 2018). Like in our study, there was no improvement in the quantity of oocytes recovered in the group with VD repletion.

Both Wu *et al*. (2018) and Antunes *et al.* (2018) have found a direct correlation between VD follicular and plasma levels. However, while Wu *et al*. (2018) encountered a significant higher number of mature oocytes and blastocyst formation rate in the higher follicular VD group, Antunes *et al*. found no significant difference between the number of mature oocytes in both groups and did not evaluate blastocysts, whilst a higher number of larger follicles (>16mm diameter) were found in the lower follicular VD group (Antunes *et al*., 2018).

It is important to emphasize that in our series, we had only 5 cases of PCOS, since some authors, such as Várbíró *et al*. (2022) and Hu *et al*. (2020), have associated this pathology with a spontaneous decrease in the chance of ovulation. In these patients, the probability of ovulation correlates with the level of VD, being 68% in cases of deficiency, 77% if insufficiency and 78% when the level is normal (Hu *et al*., 2020). We stipulate that this fact is due to the apparent relationship between the normal level of VD and the improvement in insulin resistance, present in this pathology (Hu *et al*., 2020; Shahrokhi *et al*., 2016).

In a retrospective analysis, Ko *et al*. (2022) found a direct correlation between lower cumulative live birth rates of a single IVF stimulation cycle and deficient plasma 25OH-D levels prior to ovarian stimulation. Despite that, the total number of retrieved oocytes and mature oocytes was similar between both groups, which is the same finding reported in our current research.

Some studies show that patients with VD deficiency are less successful with IVF and report more frequent miscarriages and lower live birth rates (Várbíró *et al*., 2022; Bezerra Espinola *et al*., 2021; Cyprian *et al*., 2019). Zhao *et al.* (2018) performed a meta-analysis encompassing 9 studies, reinforcing this data, since they found a higher rate of live births in patients with normal VD. However, in a more recent meta-analysis, Cozzolino *et al*. (2020) found no correlation between IVF clinical outcomes and serum VD levels. Also, Zhou *et al.* (2022) showed that there is no benefit in performing vitamin D replacement for subsequent treatment.

The strengths of this study include the specific analysis of VD influence over follicle maturation in IVF cycles, as well as the number of subjects analyzed. There is still a lot of controversies on which parts of the fertilization process VD might play a significant role, and, with this study design we could evaluate its role directly on follicular and oocyte development in ovarian stimulation cycles. Another important point to highlight is that several common confounders that could influence our results were balanced between our two groups, which was the case with obesity, age and AMH.

Despite its strengths, it is important to highlight the potential weaknesses in our study, mainly the retrospective nature of the design, as well as the lack of report on clinical outcomes, such as implantation rates, clinical pregnancy rates or even live birth rates. Although, as explained before, our focus was specifically on VD influence on the oocyte and follicle to avoid potential biases.

Another important point to emphasize is the chosen cutoff values of serum VD according to those from the Endocrine Society of North America, and not those from the Institute of Medicine, which would be higher or lower than 20ng/mL (Ross *et al*., 2011). In our study population, most patients supplemented vitamin D previously before arriving at our center for IVF treatment start. This could be an explanation for almost no patients having presented serum VD levels below 20ng/mL before ovarian stimulation began, both study groups presented average levels higher than 20ng/mL. Specific analysis comparing a group with lower than 20ng/mL serum VD with another with higher than 30ng/mL serum VD might have shown different results. However, at least in a Brazilian population, we found it difficult to enroll patients with a deficient state of serum vitamin D (<20ng/mL) before starting IVF treatment.

Regarding patients using VD supplementation, it should be noted that all VD samples were collected up to 3 months from the starting day of the ovarian stimulation onset date. Although this is another potential bias for the study, we understand that its analysis was performed in proximity to the IVF cycle and could very well reflect the actual VD plasma levels of the patients during the stimulation period. To mitigate potential alterations of the VD plasma levels from the time that VD was evaluated, and there were no changes on already started supplementations, neither were any type of supplementation started after the blood sample was collected.

## CONCLUSIONS

The results of our study showed no difference among number of follicles, oocytes retrieved, and mature oocytes obtained after the ovarian stimulation, when VD plasma levels of 30ng/mL were predefined as the benchmark values for optimal reproductive outcomes. However, VD may act in other stages of fertilization, such as implantation rates, clinical pregnancy rates, as well as, live birth rates, or even in the studied outcomes if groups with lower plasma levels were to be compared. Therefore, further studies on the subject and better evaluation of the IVF process with different VD cutoff values should be pursued.
